# Knowledge, attitude, and practice toward self-control of dental plaque among patients with periodontal diseases: a cross-sectional study

**DOI:** 10.1186/s12903-023-03352-w

**Published:** 2023-09-02

**Authors:** Jing Sun, Dongdong Tong, Chen Sun, Xin Wang, Zhibin Zuo, Yufeng Liu, Liangyan Qi, Lingxue Kong, Xiao Luan, Junru Meng

**Affiliations:** 1 Department of Periodontology, Central Laboratory, Jinan Key Laboratory of Oral Tissue Regeneration, Jinan Stomatological Hospital, Jinan, 250000 China; 2 Department of Oral and Maxillofacial Surgery, School and Hospital of Stomatology, Cheeloo College of Medicine, Shandong University; Shandong Key Laboratory of Oral Tissue Regeneration; Shandong Engineering Laboratory for Dental Materials and Oral Tissue Regeneration; Shandong Provincial Clinical Research Center for Oral Diseases, Jinan, 250012 China; 3 Department of Pharmacy, Jinan Vocational College of Nursing, Jinan, 250100 China; 4 Hospital Infection Management Office, Jinan Stomatological Hospital, Jinan, 250000 China

**Keywords:** Periodontal disease, Cross-sectional studies, Delivery of health care, Dental plaque, Gingivitis

## Abstract

**Background:**

The development of periodontal disease is closely linked to individual oral healthcare behaviors. This study aimed to investigate the knowledge, attitude, and practice (KAP) toward the self-control of dental plaque among patients with periodontal diseases.

**Methods:**

This cross-sectional study was conducted at Jinan Stomatological Hospital between July 2022 and September 2022 through a self-administrated questionnaire for patients with periodontal diseases.

**Results:**

A total of 563 participants were included. Among them, 147 (26.11%) had gingivitis and 416 (73.89%) had periodontitis. Participants' knowledge, attitude, and practice scores were 8.71 ± 2.81 (range 0–12), 39.82 ± 3.69 (range 10–50), 33.13 ± 5.91 (range 11–55), respectively. The multivariate logistic regression analysis showed that the knowledge [odds ratio (OR) = 1.212, 95% confidence interval (CI): 1.097–1.339, *P* < 0.001], attitude (OR = 1.132, 95% CI: 1.070–1.198, *P* < 0.001), occupation, especially in the commercial and service industry (OR = 0.488, 95% CI: 0.221–1.080, *P* = 0.007), and income of 10,000–20,000 yuan (OR = 0.476, 95% CI: 0.258–0.877, *P* = 0.017) were independently associated with good practice.

**Conclusions:**

Chinese patients with periodontal diseases demonstrated satisfactory knowledge and attitudes regarding oral hygiene, but the practical aspects need more promotion and training, especially in daily brushing frequency, usage of oral irrigator and interdental brush. Individualized approach should consider patients' knowledge, attitudes, occupation and income level.

**Supplementary Information:**

The online version contains supplementary material available at 10.1186/s12903-023-03352-w.

## Background

Periodontal inflammation progresses from gingival epithelium towards the periodontum, resulting in the numerous esthetic and functional problems, breakdown of the supporting periodontal tissues and tooth loss [1–3]. At present, the accepted three-factor theory of periodontitis is based on the interplay between acquired environmental factors, pathogenic oral bacteria and host factors [4, 5]. Although the development of clinically evidenced periodontitis usually takes time, aggressive forms may develop quickly, leading to the obvious decrease in the quality of life [5]. Moreover, periodontitis is the sixth most common disease worldwide, and severe periodontitis affects approximately 10% of the global population [6]. According to the Third National Oral Health Epidemiological Survey, the state of periodontal health in China is also poor, with 86% of the Chinese population suffering from varying degrees of periodontitis [7].

The treatment strategies for periodontal disease are based on the combination of periodontal therapeutic modalities to minimize symptoms and restore lost tissues, including subgingival instrumentation, local and/or systemic pharmacotherapy, and periodontal surgery [1]. The prevention of periodontitis involves, first and foremost, meticulous oral hygiene by tooth brushing, use of powered toothbrushes and interdental brushes, fluoridated toothpaste, flossing, and mouth rinses [3]. In addition, recent studies reported that uncontrolled diabetes mellitus, vitamin D insufficiency and smoking increase the susceptibility and severity of periodontitis [1, 3]. Therefore, enhancing individuals' knowledge of the protection of periodontitis and improving their oral healthcare behaviors are key initiatives in preventing and controlling periodontitis.

Knowledge, attitude, and practice (KAP) study is a structured survey method that can be applied to measure the known behaviors toward the medical conditions or preventive methods, and reveal obstacles to the activities or behavioral changes [8]. The KAP study serves as an information source and a predecessor of awareness or intervention programs to draw attention to the specific problem among the study population and beyond [9]. Despite the importance of individuals' knowledge on preventing and controlling periodontal disease, only a few KAP studies have been conducted so far. Although KAP in dentists and medical students are mostly encouraging [10, 11], in general population oral hygiene practices need to be improved [12]. To the best of our knowledge no KAP studies on oral healthcare behaviors were undertaken among patients with periodontitis in China.

Therefore, this cross-sectional study aimed to investigate KAP patterns toward the self-control of dental plaques among patients with periodontitis in China.

## Methods

### Study design and participants

This cross-sectional study was conducted at Jinan Stomatological Hospital between July 1, 2022, and September 30, 2022. Periodontitis was defined according to the new International Classification of Periodontal Diseases [13], based on the 2017 World Workshop on the Classification of Periodontal and Peri-Implant Diseases and Conditions [14]. Patients with periodontitis aged 16–70 years, who understood the purpose of the study and volunteered to participate were included, while incomplete questionnaires were excluded. This study was ethically approved by the medical ethics committee of Jinan Stomatological Hospital (No.JNSKQYY-2022–018), and informed consent was obtained from all participants.

### Procedures

This questionnaire was based on the Medical Staff Manual of *Oral Health Behaviour Guidelines for Chinese Residents* and the consensus of Chinese multidisciplinary experts on maintaining periodontal health [15], as well as previous studies accessing knowledge and practice of oral hygiene [16, 17] and revised with reference to comments made by three experts in periodontitis. The final version of questionnaire was distributed in Chinese and contained four dimensions, as follows: (1) demographic information about the participants; (2) knowledge assessment based on 12 questions about periodontal disease and control of dental plaques, scored 1 point for correct answers and 0 points for incorrect or unclear answers; total score ranging from 0 to 12 points; (3) attitude toward the self-control of dental plaques based on 10 questions, all using 5-point Likert scale, ranging from very positive (5) to very negative (1); total score ranging from 10 to 50 points; (4) practice among patients with periodontitis toward the self-control of dental plaques, based on 11 questions, also using 5-point Likert scale, ranging from always (5) to never (1), with a score range of 11–55 points.

The questions in this study showed high internal consistency with Cronbach’s *α* = 0.860 and Kaiser–Meyer–Olkin (KMO) = 0.8614. Hardcopy questionnaires were distributed in the diagnostic room and completed by patients; the clinical information was also collected at the same time. The web-based questionnaire was created and distributed using Questionnaire Star (Changsha Ranxing Information Technology Co., Ltd), which patients scanned to generate a quick response (QR) code to participate in this study. Considering that some of the patients were uncomfortable using cell phones to respond, a hardcopy version of the questionnaire was prepared and filled out by the patients after a trained research assistant explained the purpose and content of the study. All questionnaires were checked for completeness, consistency, and validity by the members of the research team.

### Statistical analysis

SPSS 26.0 (IBM Corp., NY, USA) was used for statistical analysis. Continuous data were expressed as mean ± standard deviation (SD) and compared by t-test. The categorical data were expressed as *n* (%) and compared with the chi-square test. Pearson’s correlation was used to analyze the correlations between knowledge scores, attitude scores, and practice scores. The univariable and multivariable logistic regression analyses were used to analyze the factors influencing practice; 70% of the practice score was used as the cut-off value. All statistical tests were performed using two-sided tests, and *P* values < 0.05 indicated statistically significant differences.

## Results

A total of 563 questionnaires were finally enrolled in this study, and the participants' knowledge scores were 8.71 ± 2.81 (range 0–12), attitude scores were 39.82 ± 3.69 (range 10–50), and practice scores were 33.13 ± 5.91 (range 11–55). Among them, 147 (26.11%) participants had gingivitis and 416 (73.89%) had periodontitis; the female participants accounted for 57.55%. As demonstrated in Table 1, most participants were from urban areas (92.54%), smoked (84.37%). Among them, 7.10% of the participants were younger than 20 years and 17.94% were older than 50 years. Almost a quarter of participants reported the frequency of toothbrushing per day as 0–1 times (24.16%), while others brushed their teeth 2 times or more.

**Table 1:** Baseline characteristics and knowledge, attitude, and practice (KAP) scores of study participants

Variables	*N* (%)	Knowledge		Attitude		Practice	
		Mean ± SD	*P*	Mean ± SD	*P*	Mean ± SD	*P*
**Total**		8.71 ± 2.81		39.82 ± 3.69		33.13 ± 5.91	
**Periodontal disease**			0.706		0.076		0.728
Gingivitis	147 (26.11)	8.79 ± 2.78		40.28 ± 3.76		33.28 ± 5.13	
Periodontitis	416 (73.89)	8.69 ± 2.82		39.65 ± 3.65		33.08 ± 6.17	
**Status of consultation**			< 0.001		< 0.001		< 0.001
Initial consultation	422 (74.96)	8.45 ± 2.90		39.49 ± 3.60		32.13 ± 5.43	
Secondary consultation	141 (25.04)	9.49 ± 2.37		40.80 ± 3.76		36.13 ± 6.27	
**Secondary consultation**							
Periodontitis, within 3 months of basic treatment	16 (2.84)						
Periodontitis, no consultation for at least 1 year after basic treatment and consultation	28 (4.97)						
Periodontitis, undergoing basic treatment (subgingival scaling)	49 (8.70)						
Periodontitis, annual scheduled check up	35 (6.22)						
Gingivitis, scheduled check up	13 (2.31)						
**Sex**			0.001		0.164		0.034
Male	239 (42.45)	8.23 ± 3.12		39.56 ± 3.84		32.52 ± 5.57	
Female	324 (57.55)	9.07 ± 2.50		40.00 ± 3.57		33.59 ± 6.12	
**Age (year)**			0.012		0.027		0.653
< 20	40 (7.10)	8.10 ± 2.55		41.58 ± 4.52		32.30 ± 5.94	
21–30	163 (28.95)	9.10 ± 2.52		40.15 ± 3.47		33.01 ± 5.55	
31–40	152 (27.00)	9.07 ± 2.74		39.56 ± 3.85		33.40 ± 5.57	
41–50	107 (19.01)	8.50 ± 2.78		39.35 ± 3.20		33.67 ± 6.25	
≥ 50	101 (17.94)	8.03 ± 3.30		39.47 ± 3.72		32.68 ± 6.59	
**Residence**			0.123		0.508		0.884
Rural area	42 (7.46)	8.07 ± 3.37		39.45 ± 3.58		33.26 ± 6.59	
Urban area	521 (92.54)	8.77 ± 2.76		39.84 ± 3.70		33.12 ± 5.86	
**Education**			< 0.001		0.106		0.001
High school/Technical secondary school/Below	87 (15.45)	6.87 ± 3.40		39.06 ± 3.96		30.92 ± 6.22	
junior college/University	358 (63.59)	9.06 ± 2.56		39.92 ± 3.73		33.57 ± 6.04	
graduate/Above	118 (20.96)	9.02 ± 2.53		40.06 ± 3.29		33.43 ± 4.87	
**Occupation**			0.103		0.225		0.083
Heads of party-masses organization of state organs, heads of enterprises and institutions	58 (10.30)	8.72 ± 3.05		39.21 ± 3.07		34.00 ± 5.37	
Professional and technical staff	170 (30.20)	9.19 ± 2.48		39.68 ± 3.42		33.72 ± 5.57	
Office staff, agency staff, and related staff	32 (5.68)	8.41 ± 3.08		38.91 ± 3.03		32.56 ± 4.40	
Commercial and service industry personnel	71 (12.61)	8.65 ± 3.11		40.17 ± 4.05		33.82 ± 6.12	
Others	232 (41.21)	8.43 ± 2.81		40.09 ± 3.95		32.35 ± 6.32	
**Income (yuan)**			0.113		0.086		0.017
< 5000	114 (20.25)	8.16 ± 3.23		39.57 ± 4.02		32.75 ± 6.35	
5000–10,000	245 (43.52)	8.79 ± 2.80		39.76 ± 3.58		33.29 ± 5.82	
10,000–20,000	158 (28.06)	8.91 ± 2.54		39.69 ± 3.54		32.47 ± 5.46	
> 20,000	46 (8.17)	9.02 ± 2.53		41.13 ± 3.75		35.52 ± 6.25	
**Marital status**			0.03		0.001		0.84
Unmarried	191 (33.93)	9.07 ± 2.35		40.57 ± 3.71		33.06 ± 5.67	
Married	372 (66.07)	8.53 ± 3.00		39.43 ± 3.62		33.17 ± 6.04	
**Underlying diseases**			0.801		0.421		0.302
Have	511 (90.76)	8.70 ± 2.86		39.86 ± 3.74		33.22 ± 5.84	
None	52 (9.24)	8.81 ± 2.23		39.42 ± 3.06		32.33 ± 6.52	
**Smoking**			0.027		0.046		0.712
Never smoked	475 (84.37)	8.85 ± 2.77		39.98 ± 3.72		33.19 ± 6.02	
Previously smoked	43 (7.64)	7.95 ± 2.72		38.98 ± 3.24		32.42 ± 5.04	
Still smoking now	45 (7.99)	8.00 ± 3.13		38.87 ± 3.58		33.18 ± 5.54	
**Times of drinking (per month)**			0.031		0.514		0.7
0 times	370 (65.72)	8.84 ± 2.75		39.94 ± 3.73		33.13 ± 6.33	
1–5 times	166 (29.48)	8.66 ± 2.70		39.62 ± 3.50		33.00 ± 5.06	
6–10 times	27 (4.80)	7.37 ± 3.86		39.33 ± 4.22		34.04 ± 4.69	
**Times of toothbrushing (per day)**			< 0.001		< 0.001		< 0.001
0–1 times	136 (24.16)	7.69 ± 3.18		38.86 ± 3.58		30.24 ± 5.46	
2–3 times	411 (73.00)	9.04 ± 2.61		40.04 ± 3.67		33.90 ± 5.60	
> 3 times	16 (2.84)	8.94 ± 2.57		42.13 ± 3.20		38.00 ± 8.10	

**Fig. 1:**
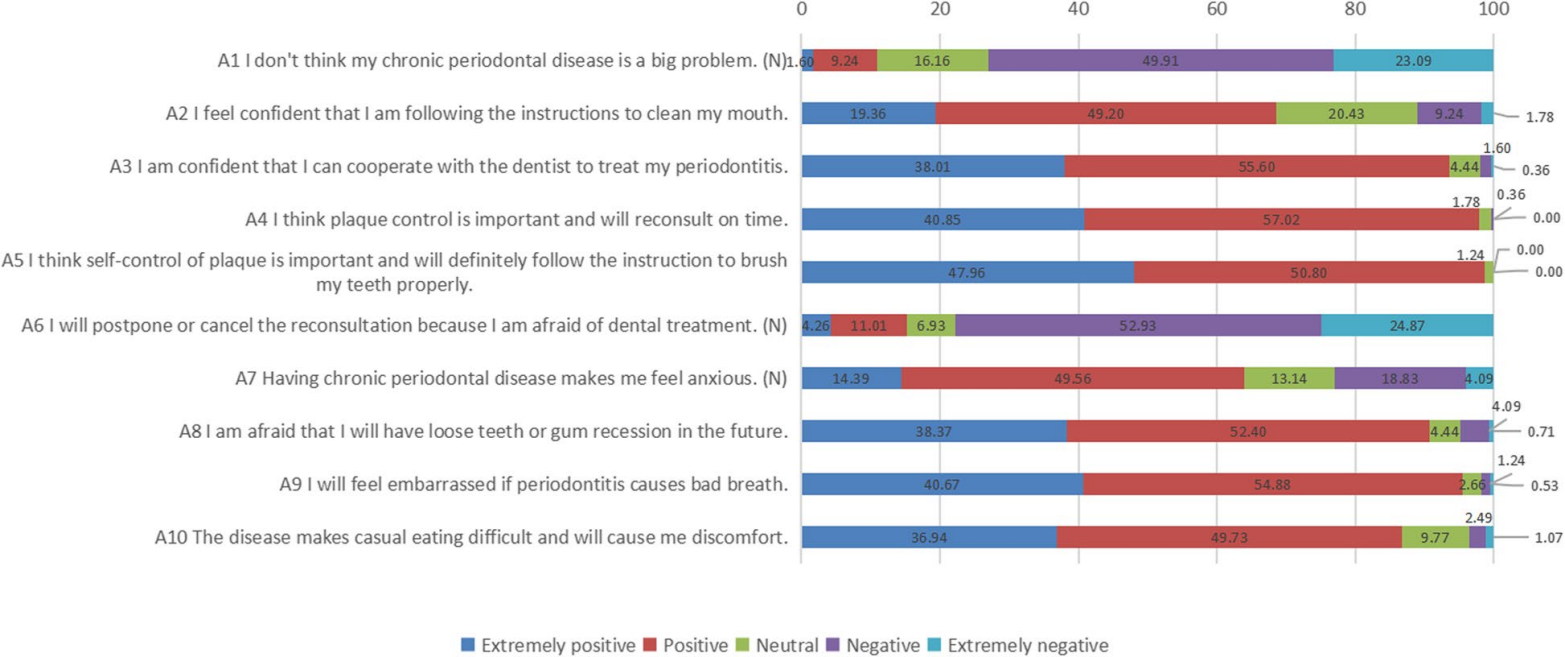
Distribution of answers in the “attitude” dimension. (N) means that the statement of the question stem is negative, unlike other questions, and uses reverse assignment of marks

**Fig. 2:**
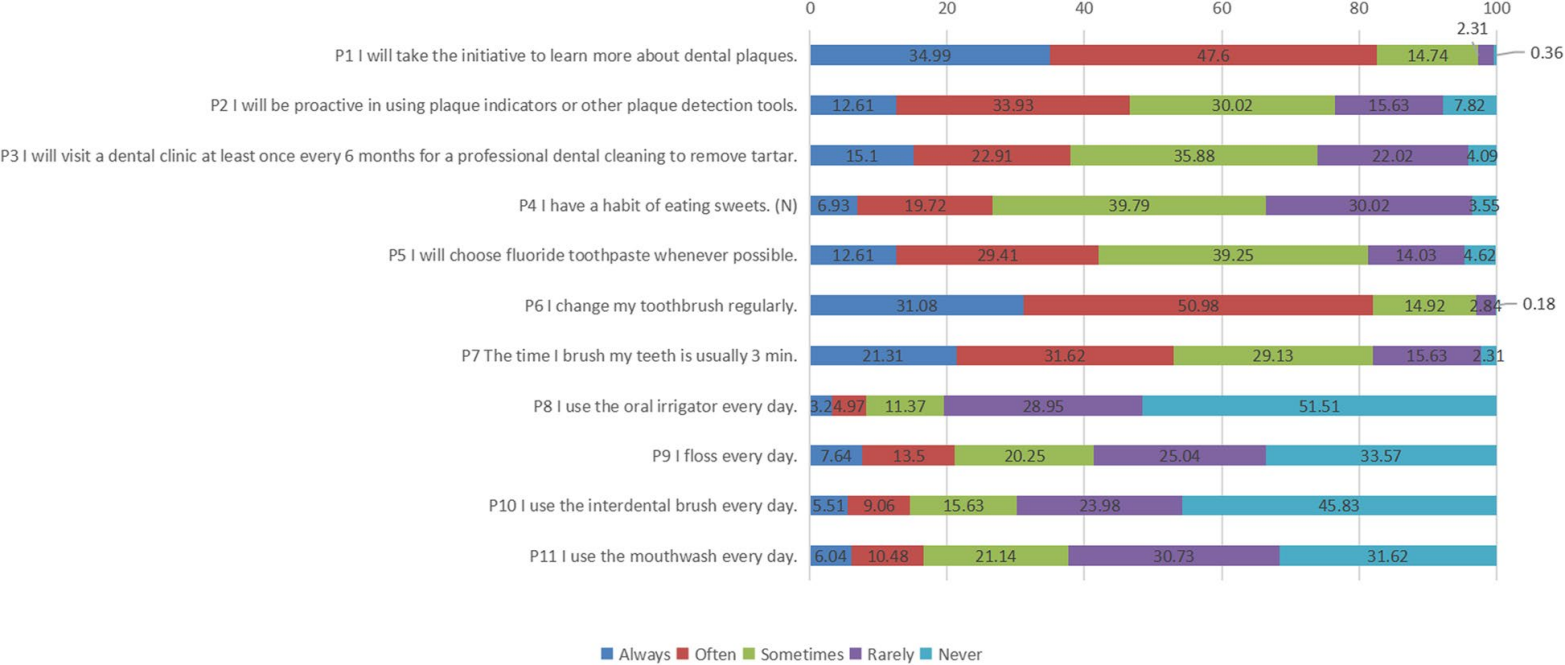
Distribution of answers in the “practice” dimension. (N) means that the statement of the question stem is negative, unlike other questions, and uses reverse assignment of marks

In the knowledge dimension (Table 2), the most correctly answered question was about the minimal number of toothbrushings needed per day (90.76% of the participants answered correctly), while the least correct was the “Bass method” (53.82%). In the attitude dimension (Fig. 1), the participants demonstrated a positive attitude toward the “self-control of plaque” aspect (98.80%), while a small number reported feeling anxious toward their periodontitis (22.90%). In the practice dimension (Fig. 2), the initiative for self-learning was the most notable (82.60%), while the use of an oral irrigator was the least frequent practice (8.20%).

**Table 2:** Detailed answers of study participants in Knowledge dimension

Knowledge	*N* (%)	
	Correct	Wrong/unclear
1. Periodontitis is caused by bacteria in dental plaque	427 (75.84)	136 (24.16)
2. Swelling and bleeding gums are early signs of bacterial infection of the gums	445 (79.04)	118 (20.96)
3. Plaque can build up on your teeth if your oral hygiene becomes poor or without a regular dental check-up	483 (85.79)	80 (14.21)
4. Plaque can increase the risk of tooth decay or gum disease	479 (85.08)	84 (14.92)
5. If the dental plaque is not removed daily (such as by brushing), it can harden or calcify and become tartar, also known as dental calculus	489 (86.86)	74 (13.14)
6. To remove plaque between the teeth and under the teeth ridge, it is important to floss your teeth daily, as plaque in these areas is difficult to remove with a toothbrush	372 (66.07)	191 (33.93)
7. To remove plaque from all surfaces of the teeth, brush your teeth at least twice a day	511 (90.76)	52 (9.24)
8. People cannot prevent the formation of plaque but can remove it actively, which also minimizes the formation of dental calculus	475 (84.37)	88 (15.63)
9. Limiting the intake of sugary foods can also help control the formation of plaque, as it can reduce the reproduction of bacteria in the mouth	479 (85.08)	84 (14.92)
10. In the Bass method, the toothbrush is placed on the thumb and index finger, and the toothbrush bristles are aimed at the junction of teeth and gums because plaque is concentrated here, and the aim of brushing is to clean the plaque	303 (53.82)	260 (46.18)
11. The lingual side of the back teeth of the upper jaw is a more difficult part to brush; therefore, it is more important to pay attention to cleaning this part	443 (78.69)	120 (21.31)
12. Flossing is an important means to clean tooth gaps and cannot be replaced by brushing and rinsing the mouth	424 (75.31)	139 (24.69)

Pearson correlation analysis showed that the correlation between knowledge and practice (*r* = 0.313, *P* < 0.001). In contrast, the correlations between attitude and knowledge (*r* = 0.287, *P* < 0.001) as well as attitude and practice (*r* = 0.275, *P* < 0.001) were slightly less strong (Table 3).

**Table 3:** Pearson correlation analysis

	Knowledge	Attitude	Practice
Knowledge	1		
Attitude	0.287 (*P* < 0.001)	1	
Practice	0.313 (*P* < 0.001)	0.275 (*P* < 0.001)	1

The multivariate logistic regression analysis showed that the knowledge (OR = 1.212, 95% CI: 1.097–1.339, *P* < 0.001) and attitude (OR = 1.132, 95% CI: 1.070–1.198, *P* < 0.001) were independently associated with good practice. At the same time, occupation, such as the commercial and service industry (OR = 0.488, 95% CI: 0.221–1.080, *P* = 0.007) and with income level of 10,000–20,000 yuan (OR = 0.476, 95% CI: 0.258–0.877, *P* = 0.017) were the independent predictors of poor practice. Although the education level was also shown to be linked to better practice patterns by the univariate analysis, the multivariate analysis did not confirm statistical significance (*P* = 0.407). Finally, among other habits, better practice patterns were linked to the frequency of toothbrushing 2–3 times per day (OR = 1.697, 95% CI: 1.013–2.844, *P* = 0.044) and  > 3 times per day (OR = 5.742, 95% CI: 1.688–19.530, *P* = 0.005) (Table 4). Subgroup analyses were performed considering the differences in consultation status, taking into account the relatively stable conditions, patient education, and good compliance observed among the follow-up patients who had undergone secondary consultation, in comparison to those who had received initial consultation (Supplementary Table 1 & Supplementary Table 2).

**Table 4:** Factors influencing practice patterns among study participants

Factors	Univariate logistic regression		Multivariate logistic regression	
	OR (95% CI)	*P*	OR (95% CI)	*P*
Knowledge score	1.293 (1.177–1.421)	< 0.001	1.212 (1.097–1.339)	< 0.001
Attitude score	1.166 (1.107–1.227)	< 0.001	1.132 (1.070–1.198)	< 0.001
**Periodontal disease**				
Gingivitis	Ref	-		
Periodontitis	1.077 (0.717–1.617)	0.72		
**Status of consultation**				
Initial consultation	ref	-		
Secondary consultation	1.520 (1.342–1.722)			
**Sex**				
Male	Ref	-		
Female	1.224 (0.853–1.757)	0.273		
**Age (year)**				
< 20	Ref	-		
21–30	1.613 (0.736–3.536)	0.232		
31–40	1.108 (0.498–2.467)	0.802		
41–50	1.586 (0.699–3.597)	0.27		
≥ 50	1.522 (0.666–3.478)	0.319		
**Residence**				
Rural area	Ref	-		
Urban area	1.043 (0.529–2.058)	0.903		
**Education**				
High School/Technical secondary school/Below	Ref	-	Ref	-
junior college/University	2.209 (1.246–3.917)	0.007	1.328 (0.679–2.595)	0.407
graduate/Above	1.881 (0.975–3.629)	0.06	0.961 (0.436–2.119)	0.922
**Occupation**				
Heads of party-masses organization of state organs, heads of enterprises and institutions	Ref	-	Ref	-
Professional and technical staff	0.626 (0.342–1.146)	0.129	0.481 (0.247–0.938)	0.032
Office staff, agency staff, and related staff	0.321 (0.120–0.860)	0.024	0.272 (0.092–0.800)	0.018
Commercial and service industry personnel	0.624 (0.307–1.269)	0.193	0.488 (0.221–1.080)	0.007
Others	0.401 (0.221–0.725)	0.003	0.287 (0.144–0.569)	< 0.001
**Income (yuan)**				
< 5000	Ref	-	Ref	-
5000–10,000	0.899 (0.560–1.445)	0.661	0.592 (0.342–1.026)	0.062
10,000–20,000	0.724 (0.428–1.225)	0.229	0.476 (0.258–0.877)	0.017
> 20,000	2.000 (0.996–4.015)	0.051	1.252 (0.563–2.784)	0.582
**Marital status**				
Unmarried	Ref	-		
Divorced	0.921 (0.634–1.337)	0.664		
**Underlying diseases**				
Yes	0.858 (0.458–1.607)	0.632		
No	ref	-		
**Smoking**				
Never smoked	Ref	-		
Previously smoked	0.839 (0.419–1.679)	0.619		
Still smoking now	1.315 (0.699–2.477)	0.396		
**Times of drinking**				
0	Ref	-		
1–5	0.921 (0.619–1.369)	0.683		
≥ 6–10	1.468 (0.661–3.262)	0.346		
**Times of teeth brushing**				
0–1	Ref	-	Ref	-
2–3	2.395 (1.483–3.866)	< 0.001	1.697 (1.013–2.844)	0.044
> 3	7.400 (2.460–22.258)	< 0.001	5.742 (1.688–19.530)	0.005

The comparison of KAP patterns between patients with gingivitis and periodontitis is demonstrated in Table 5. There were no significant differences in most knowledge and practices, while, in the attitude dimension, the notion of postponing or canceling the consultation due to the fear of dental treatment was more typical for patients with chronic gingivitis (*P* = 0.038).

**Table 5:** Differences in knowledge, attitude, and practice (KAP) scores between study populations

Factor or statement	Participants		*P*
	Gingivitis	Periodontitis	
Knowledge	8.79 ± 2.78	8.69 ± 2.82	0.706
Attitude			
Total	40.28 ± 3.76	39.65 ± 3.65	0.076
A1 I don’t think my periodontitis is a big problem	3.87 ± 0.97	3.82 ± 0.93	0.608
A6 Postponing or canceling the consultation because I am afraid of dental treatment	3.99 ± 1.00	3.78 ± 1.07	0.038
Practice			
Total	33.28 ± 5.13	33.08 ± 6.17	0.728
P2 Plaque detection tools	3.35 ± 0.98	3.25 ± 1.15	0.388
P3 Dental cleaning	3.34 ± 1.06	3.19 ± 1.08	0.147
P5 Fluoride toothpaste	3.27 ± 1.00	3.33 ± 1.02	0.557
P6 Toothbrush changing	4.10 ± 0.78	4.10 ± 0.76	0.962
P7 Enough brushing time	3.51 ± 1.02	3.55 ± 1.08	0.693
P8 Using an oral irrigator daily	1.81 ± 0.98	1.79 ± 1.06	0.833
P9 Flossing teeth daily	2.38 ± 1.26	2.36 ± 1.29	0.868
P10 Using interdental brush daily	1.97 ± 1.13	2.07 ± 1.24	0.406
P11 Using mouthwash daily	2.39 ± 1.12	2.25 ± 1.21	0.198

## Discussion

This study found that the knowledge and attitudes toward oral hygiene among patients with periodontitis in China was satisfactory while practice may need to be improved. In addition to knowledge and attitudes, patients' occupation, income level, and daily brushing frequency may also influence their practice. These results might lead to divergence in periodontitis situation and changes in oral hygiene habits in patients who are aware of their diagnosis.

Knowledge and practice of oral hygiene in China are relatively poor, especially among 35- to 45-year-old and 65- to 74-year-old groups [18, 19]. This study included all age groups, and although knowledge and attitude differed slightly in different age groups, age was not associated with better practice scores. With the majority of participants demonstrating satisfactory knowledge, the mean practice scores were not high (33.13 out of 55), but many oral hygiene habits were practiced more often compared to general population. In particular, previous study by Zhu et al. [20] reported that only 32% of the 35- to 44-year-olds and 23% of the 65- to 74-year-olds brushed their teeth at least twice a day, while in this study, almost three fourths of all participants reported brushing their teeth 2 times per day or more often. In the study by An et al. [19] conducted during the same period, only 26.3% of participants used fluoride toothpaste and 73.7% did not apply or did not know about fluoride toothpaste. However, in the present study, fluoride toothpaste was used often or always by 42.1% and occasionally by 39.3% of participants. These results suggested that patients with periodontitis might be more informed about oral hygiene than the general population, most likely because of the information received during previous visits to the dentist.

Some studies reported the unsatisfactory effect of oral health education as a primary preventive method in China [19, 21]. In this study, the initiative for self-learning was the most notable (82.6%), but more practical aspects still need promotion and training, such as oral irrigator (used often or always by only 8.2% of participants) or interdental brush (used often or always by 14.6%). Knowledge was linked to practice slightly more than attitude, which was in line with previous studies conducted in China [21, 22] and worldwide [16, 23]. In patients who belonged to the risk groups for periodontitis more positive attitudes and proactive seeking for more oral health knowledge might lead to better practice [23, 24]. On the other hand in this study a positive attitude was more likely found in the answers that were impossible to check, and the actual behavior might differ. This discrepancy between actual and reported attitude should be taken into account in the future studies.

According to the most recent national health survey, Chinese adults who seek dental care services already have poor oral conditions and need treatment rather than prevention [25]. In this study, occupations such as commercial and service industry were independent factors related to better practice, suggesting that more frequent human contact needed in daily jobs might play an important role for Chinese adults, influencing better practice. At the same time, despite recent reports [26, 27] that participants with higher education levels tended to have better oral health hygiene, the multivariate analysis in this study did not confirm statistically significant correlations between education level and better practice patterns (*P* = 0.407) for the study population. As noted earlier, these differences were most likely explained by the knowledge obtained during the previous visits to the dentist, as well as the predominantly urban population in this study. Thus, the educational interventions carried out by dentists for the patients with periodontitis have the potential to promote the self-control of dental plaques in the population.

This study had some limitations. First, the cross-sectional design did not allow for concluding causal relationships; the questionnaire was self-designed, leading to lower applicability of the obtained results. Second, noticeable differences might exist between the reported and actual behaviors, as participants filled out the questionnaire in the hospital and thus might naturally want to appear more conscious and knowledgeable. Finally, although the study population was not small, most participants lived in urban areas; the oral hygiene practice and periodontitis prevention practice were reportedly worse in rural areas. These limitations should be taken into account while interpreting the aforementioned results and planning future studies.

## Conclusion

In conclusion, this study found that Chinese patients with periodontitis demonstrated satisfactory knowledge and attitudes about plaque control, but the practical aspects, such as usage of oral irrigator and interdental brush need more promotion and training. Patients’ knowledge, attitudes, occupation, income level, and daily brushing frequency may influence their practice. These findings might contribute to the ongoing effort of enhancing individuals’ knowledge of oral hygiene and other key initiatives in preventing and controlling periodontitis in China and worldwide.

### Supplementary Information

**Additional file 1:** **Supplementary Table 1. **Subgroup analysis for initial consultation.

**Additional file 2:** **Supplementary Table 2. **Subgroup analysis for secondary consultation.

## Data Availability

All data generated or analyzed during this study are included in this published article.

## Abbreviations

KAP: Knowledge, attitude, and practice
KMO: Kaiser-Meyer-Olkin
QR: Quick response
SD: Standard deviation
