# Supplementary material for: Knowledge, attitude, and practice toward self-control of dental plaque among patients with periodontal diseases: a cross-sectional study
Source: BMC Oral Health. 2023 Sep 2;23:628. doi: 10.1186/s12903-023-03352-w (PMC10475179; doi:10.1186/s12903-023-03352-w)
Supplement: Supplementary file 1 — Additional file 1: Supplementary Table 1. Subgroup analysis for initial consultation. [file 12903_2023_3352_MOESM1_ESM.docx]

**Supplementary Table 1.** Subgroup analysis for initial consultation

| Factors | Univariate logistic regression | | Multivariate logistic regression | |
| --- | --- | --- | --- | --- |
|  | OR (95% CI) | *P* | OR (95% CI) | *P* |
| Knowledge score | 1.253(1.117-1.406) | ＜0.001 | 1.187(1.052-1.34) | 0.005 |
| Attitude score | 1.157(1.084-1.234) | ＜0.001 | 1.129(1.05-1.213) | 0.001 |
| **Periodontal disease** |  |  |  |  |
| Gingivitis | Ref | - |  |  |
| Periodontitis | 0.704(0.439-1.13) | 0.146 |  |  |
| **Sex** |  |  |  |  |
| Male | Ref | - |  |  |
| Female | 1.067(0.673-1.692) | 0.783 |  |  |
| **Age (year)** |  |  |  |  |
| <20 | Ref | - |  |  |
| 21–30 | 2.381(0.859-6.601) | 0.095 |  |  |
| 31–40 | 1.243(0.431-3.586) | 0.688 |  |  |
| 41–50 | 2.109(0.717-6.2) | 0.175 |  |  |
| ≥50 | 1.604(0.518-4.969) | 0.413 |  |  |
| **Residence** |  |  |  |  |
| Rural area | Ref | - |  |  |
| Urban area | 1.04(0.434-2.492) | 0.93 |  |  |
| **Education** |  |  |  |  |
| High School/Technical secondary school/Below | Ref | - | Ref | - |
| junior college/University | 3.662(1.516-8.843) | 0.004 | 2.239(0.86-5.827) | 0.099 |
| graduate/Above | 3.818(1.464-9.961) | 0.006 | 1.9(0.646-5.586) | 0.244 |
| **Occupation** |  |  |  |  |
| Heads of party-masses organization of state organs, heads of enterprises and institutions | 2.826(1.313-6.082) | 0.008 | 3.828(1.593-9.2) | 0.003 |
| Professional and technical staff | 1.449(0.836-2.513) | 0.186 | 1.273(0.693-2.339) | 0.436 |
| Office staff, agency staff, and related staff | 0.888(0.285-2.767) | 0.838 | 1.006(0.304-3.323) | 0.993 |
| Commercial and service industry personnel | 1.85(0.912-3.756) | 0.088 | 1.984(0.906-4.344) | 0.087 |
| Others | Ref | - | Ref | - |
| **Income (yuan)** |  |  |  |  |
| <5000 | 1.202(0.639-2.262) | 0.569 | 0.49(0.189-1.269) | 0.142 |
| 5000–10,000 | 0.894(0.442-1.81) | 0.755 | 0.358(0.158-0.814) | 0.014 |
| 10,000–20,000 | 3.319(1.416-7.779) | 0.006 | 0.243(0.101-0.584) | 0.002 |
| >20,000 | Ref | - | Ref | - |
| **Marital status** |  |  |  |  |
| Unmarried | Ref | - |  |  |
| Divorced | 0.869(0.545-1.384) | 0.554 |  |  |
| **Underlying diseases** |  |  |  |  |
| Yes | 1.507(0.607-3.743) | 0.377 |  |  |
| No | Ref | - |  |  |
| **Smoking** |  |  |  |  |
| Never smoked | Ref | - |  |  |
| Previously smoked | 0.803(0.294-2.196) | 0.669 |  |  |
| Still smoking now | 1.467(0.671-3.205) | 0.337 |  |  |
| **Times of drinking** |  |  |  |  |
| 0 | Ref | - |  |  |
| 1–5 | 1.103(0.668-1.821) | 0.701 |  |  |
| ≥6–10 | 2.187(0.692-6.918) | 0.183 |  |  |
| **Times of teeth brushing** |  |  |  |  |
| 0–1 | Ref | - | Ref | - |
| 2–3 | 1.959(1.112-3.448) | 0.02 | 1.434(0.777-2.646) | 0.249 |
| ＞3 | 5.667(1.488-21.577) | 0.011 | 4.067(0.907-18.233) | 0.067 |
