# Supplementary material for: Knowledge, attitude, and practice toward self-control of dental plaque among patients with periodontal diseases: a cross-sectional study
Source: BMC Oral Health. 2023 Sep 2;23:628. doi: 10.1186/s12903-023-03352-w (PMC10475179; doi:10.1186/s12903-023-03352-w)
Supplement: Supplementary file 2 — Additional file 2: Supplementary Table 2. Subgroup analysis for secondary consultation. [file 12903_2023_3352_MOESM2_ESM.docx]

**Supplementary Table 2.** Subgroup analysis for secondary consultation

| Factors | Univariate logistic regression | | Multivariate logistic regression | |
| --- | --- | --- | --- | --- |
|  | OR (95% CI) | *P* | OR (95% CI) | *P* |
| Knowledge score | 1.237(1.054-1.452) | 0.009 | 1.17(0.986-1.389) | 0.072 |
| Attitude score | 1.132(1.029-1.246) | 0.011 | 1.1(0.993-1.218) | 0.068 |
| **Status of consultation** |  |  |  |  |
| Initial consultation | Ref | - |  |  |
| Secondary consultation | 0.83(0.257-2.675) | 0.754 |  |  |
| **Sex** |  |  |  |  |
| Male | Ref | - | Ref | - |
| Female | 2.258(1.142-4.463) | 0.019 | 1.531(0.657-3.567) | 0.324 |
| **Age (year)** |  |  |  |  |
| <20 | Ref | - |  |  |
| 21–30 | 0.327(0.034-3.181) | 0.336 |  |  |
| 31–40 | 0.308(0.032-2.942) | 0.306 |  |  |
| 41–50 | 0.227(0.024-2.165) | 0.197 |  |  |
| ≥50 | 0.21(0.023-1.958) | 0.171 |  |  |
| **Residence** |  |  |  |  |
| Rural area | Ref | - |  |  |
| Urban area | 1.136(0.33-3.914) | 0.839 |  |  |
| **Education** |  |  |  |  |
| High School/Technical secondary school/Below | Ref | - |  |  |
| junior college/University | 0.955(0.34-2.681) | 0.93 |  |  |
| graduate/Above | 0.552(0.165-1.838) | 0.333 |  |  |
| **Occupation** |  |  |  |  |
| Heads of party-masses organization of state organs, heads of enterprises and institutions | 1.167(0.418-3.255) | 0.768 |  |  |
| Professional and technical staff | 1.562(0.669-3.646) | 0.303 |  |  |
| Office staff, agency staff, and related staff | 0.485(0.104-2.266) | 0.357 |  |  |
| Commercial and service industry personnel | 0.808(0.283-2.304) | 0.69 |  |  |
| Others | Ref | - |  |  |
| **Income (yuan)** |  |  |  |  |
| <5000 | 1.333(0.311-5.716) | 0.698 |  |  |
| 5000–10,000 | 0.673(0.179-2.541) | 0.56 |  |  |
| 10,000–20,000 | 0.602(0.151-2.39) | 0.47 |  |  |
| >20,000 | Ref | - |  |  |
| **Marital status** |  |  |  |  |
| Unmarried | Ref | - |  |  |
| Divorced | 0.62(0.286-1.344) | 0.226 |  |  |
| **Underlying diseases** |  |  |  |  |
| Yes | 1.231(0.445-3.403) | 0.689 |  |  |
| No | Ref | - |  |  |
| **Smoking** |  |  |  |  |
| Never smoked | Ref | - |  |  |
| Previously smoked | 0.47(0.167-1.326) | 0.154 |  |  |
| Still smoking now | 0.94(0.281-3.148) | 0.92 |  |  |
| **Times of drinking** |  |  |  |  |
| 0 | Ref | - | Ref | - |
| 1–5 | 0.454(0.216-0.953) | 0.037 | 0.584(0.242-1.405) | 0.229 |
| ≥6–10 | 0.389(0.123-1.232) | 0.108 | 0.739(0.195-2.797) | 0.656 |
| **Times of teeth brushing** |  |  |  |  |
| 0–1 | Ref | - |  |  |
| 2–3 | 1.774(0.619-5.084) | 0.286 |  |  |
| ＞3 | 6.429(0.605-68.312) | 0.123 |  |  |
